# Characterisation of fibronectin-mediated FAK signalling pathways in lung cancer cell migration and invasion

**DOI:** 10.1038/sj.bjc.6605154

**Published:** 2009-06-30

**Authors:** X N Meng, Y Jin, Y Yu, J Bai, G Y Liu, J Zhu, Y Z Zhao, Z Wang, F Chen, K-Y Lee, S B Fu

**Affiliations:** 1 Laboratory of Medical Genetics, Department of Biology, Harbin Medical University, Harbin 150086, China; 2 Department of Cell Biology and Anatomy, The University of Calgary, Calgary, Canada

**Keywords:** FAK, lung cancer, metastasis

## Abstract

**Background:**

Focal adhesion kinase (FAK) is overexpressed in a variety of cancers, such as breast, colon, prostate, ovary, and lung cancers. However, the mechanism by which extracellular matrix fibronectin stimulates lung cancer cell migration and invasion through FAK remains to be investigated.

**Methods:**

The signalling pathways in fibronectin-mediated lung cancer cell migration and invasion were examined using western blotting. The metastasis function was detected by wound healing, migration and invasion assays. Further, RNA interference and kinase inhibitors were also used to study the downstream signals.

**Results:**

In this study, we examined the FAK signalling pathways in relation to calpain-2 and RhoA in fibronectin-mediated lung cancer cell migration and invasion. We found that A549 lung epithelial cells stimulated by fibronectin showed increased phosphorylation of FAK and its downstream targets, Src, ERK1/2, phosphatidylinositol 3′-kinase (PI3K), and Akt. Consistent with this observation, depletion of FAK by siRNA resulted in the inhibition of Src, ERK1/2, PI3K, and Akt activity. In addition, the Src inhibitor, PP2, blocked the phosphorylation of FAK, ERK1/2, PI3K, and Akt. Conversely, inhibition of MEK1/2 using PD98059 reduced the expression of matrix metalloproteinase-9 (MMP9) and calpain-2. The PI3K inhibitor, LY294002, further blocked the expression of MMP9 and RhoA. Inhibition of both MEK1/2 and PI3K caused reduced cell migration and invasion.

**Conclusion:**

Our data suggest that fibronectin-mediated activation of FAK that leads to lung cancer metastasis could occur through ERK or PI3K/Akt regulation of MMP9/calpain-2 or MMP9/RhoA activity, respectively.

Lung cancer is one of the most common types of cancer in the world. The major health threat for lung cancer is death due to metastasis, a multi-step process requiring the ability of cancer cells to escape control of their surrounding microenvironment and invade the basement membrane. Focal adhesion kinase (FAK), a cytoplasmic kinase that is involved in extracellular matrix (ECM)/integrin-mediated signalling pathways, has been suggested to have an essential role in metastasis, through the modulation of tumour cell migration and invasion (Schaller *et al*, 1992). After integrin activation, FAK exhibits increased kinase activity through phophorylation at its Tyr397 site (Lipfert *et al*, 1992). Indeed, FAK activity was found to be upregulated in cancer cell lines and in tissue lysates obtained from patients with metastatic disease (Cance *et al*, 2000). An important role of FAK in cancer cell invasion was further supported by RNA interference (RNAi) studies (Huang *et al*, 2005) and by the use of a specific FAK inhibitor to block metastasis (van Nimwegen *et al*, 2005).

Activated FAK in cancer cells relays signals through multiple downstream targets. For example, when FAK is activated through autophosphorylation at Tyr397, it binds the Src-homology domain 2 (SH2) of phosphatidylinositol 3′-kinase (PI3K), thereby transporting the catalytic subunit of PI3K to the membrane, where it catalyzes the phosphorylation of inositol lipids at the D-3 position to form 3′-phosphorylated phosphoinositides, including phosphatidylinositol- 3,4,5-trisphosphate (Chen *et al*, 1996). The residues surrounding Tyr397 can also constitute a sequence that binds to the SH2 motif of other tyrosine kinases, such as pp60^*c*−*src*^. Binding of FAK to pp60^*c*−*src*^ also links FAK to the adaptor protein Grb2 and to the Ras signalling pathway (Schlaepfer and Hunter, 1997; Schlaepfer *et al*, 1998). The downstream targets of the Ras signalling pathway include ERK1/2 (Sanders and Basson, 2000). Indeed, these pathways are activated during integrin binding to the ECM, resulting in the transduction of external stimuli from the ECM to the nucleus (Sawai *et al*, 2005).

In cancer cells, FAK has been shown to regulate cell migration and invasion through distinct pathways by promoting the dynamic regulation of focal adhesion and peripheral actin structures (Lauffenburger and Horwitz, 1996; Friedl and Bröcker, 2000; Schlaepfer *et al*, 2004), as well as the matrix metalloproteinases (MMP)-mediated matrix degradation (Siesser and Hanks, 2006). In order to migrate, the leading edge of the cell forms peripheral adhesions, whereas the rear part of the cell breaks down into focal adhesions. On the other hand, calpain critically regulates cell motility through its involvement in the disassembly of focal adhesion. Consistent with these views, tumours that have metastasised have been found to have higher levels of calpain than those that are not metastatic (Carragher *et al*, 2003). In separate studies, RhoA has been implicated in the regulation of the contraction and retraction forces that are required for cell migration (Etienne-Manneville and Hall, 2002; Raftopoulou and Hall, 2004). Certainly, FAK-deficient fibroblasts have increased RhoA activity but re-expression of FAK in these cells decreased RhoA activity (Ren *et al*, 2000). Together, these findings concur with the observation that the metastatic capability correlates with MMP9 expression in tumour cells (Björklund and Koivunen, 2005). Indeed, the enhanced expression of MMP-9 has been associated with human lung cancer invasion and metastasis (Yang *et al*, 2005).

Although there are numerous suggestions that the ECM promotes cancer cell migration and invasion through FAK signalling, the mechanism by which ECM fibronectin regulates FAK activity in lung cancer cells, particularly in relation to calpain-2 and RhoA, has not yet been explored in detail. In this study, we propose that fibronectin-induced stimulation of FAK that causes lung cancer cell migration and invasion occurs by the activation of MMP9/calpain-2 and MMP9/RhoA through the ERK and PI3K/Akt signalling pathways, respectively.

## MATERIALS AND METHODS

### Antibodies and reagents

FAK (clone 4. 47) and p-FAK (Tyr397) antibodies were from Upstate Biologicals Inc. (New York, NY, USA). p-FAK (Tyr861) was obtained from BioSource International (Camarillo, CA, USA). p-FAK (Tyr925), p-Src (Tyr416), Src, p-PI3K p85 (Tyr458)/p55 (Tyr199), PI3K p85, p-Akt (Ser473), p-ERK1/2 (Thr202/Tyr204), and RhoA (67B9) antibodies were from Cell Signaling Technology Inc (Danvers, MA, USA). ERK2 (C-14), Akt1(C-20), MMP-9 (C-20), calpain-2 (N-19), and GAPDH (1D4) antibodies were purchased from Santa Cruz Biotechnology (Santa Cruz, CA, USA). PP2, PD98059, and LY294002 were from Calbiochem (San Diego, CA, USA). The FAK SMARTpool siRNA, control siRNA, and the transfection reagent were obtained from Dharmacon (Lafayette, CO, USA). Fibronectin from human plasma was from Sigma-Aldrich (New York City, NY, USA).

### Cell culture

The A549 human lung cancer cell line was obtained from the American Type Culture Collection (Manassas, VA, USA) and cultured in Ham's F12K medium (Sigma-Aldrich) supplemented with 10% fetal bovine serum (FBS) (Hyclone, Logan, UT, USA) at 37 °C with 5% CO_2_.

### Wound healing assay

Cells were plated into 24-well plates and grown to confluence. The monolayer was wounded using the tip of a sterile 200-*μ*l pipette (width: ∼1 mm). Cell debris were removed by washing twice with the serum-free medium. The cells were then allowed to migrate into the denuded areas for 10 h. Photomicrographs were taken immediately after wounding and following 10 h of incubation using the Nikon Diaphot TMD inverted microscope (Nikon, Chiba, Japan). The percentage (%) change in migration was determined by comparing the difference in wound width (*n*=3).

### Migration assay

Transwell plates (24-well, 8-*μ*m pore size; Costar, Cambridge, MA, USA) were used to conduct the migration assay. The lower chamber of the transwell plates were filled with 500 *μ*l of Ham's F12K medium containing 10% FBS. Cells (5 × 10^4^) suspended in Ham's F12K medium that contained 1% FBS were added to the upper chamber, and the plate was incubated at 37 °C with 5% CO_2_ for 6 h. Cells on the upper surface of the filters were removed using cotton swabs. Cells that migrated to the lower surface of the filters were washed, fixed, and stained with hematoxylin and eosin, and counted under the microscope. The percentage change in migration was determined by counting the number of cells that migrated to the lower surface of the filters. At least three separate microscopic fields were counted per membrane (*n*=3).

### Invasion assay

Cell invasion through a three-dimensional ECM was assessed using BD Matrigel invasion chambers (BD, Biocoat, Bedford, MA, USA) with 8.0-*μ*m filter membranes. Cells (5 × 10^4^) re-suspended in 500 *μ*l of Ham's F12K medium that contained 1% FBS were plated into the chamber inserts, and those re-suspended in 750 *μ*l of Ham's F12K that contained 10% FBS were placed into the lower chamber. After 24 h, cells invading the lower surface of the filters were fixed, stained, and counted. The percentage change in invasion was determined by counting the number of cells that migrated to the lower surface of the filters. At least three separate microscopic fields were counted per membrane (*n*=3).

### RNA interference

A549 lung cancer cells were transfected with FAK siRNA or control non-specific siRNA according to the manufacturer's instructions. Briefly, cells were grown in 35-mm dishes and transfected with the siRNA at a concentration of 200 pmol per well. Depletion of FAK was determined by western blotting. The functional effects of FAK on fibronectin-induced A549 cell migration and invasion were evaluated by wound healing, migration, and invasion assays as described above.

### Western blot analysis

Cells were harvested and solubilised in the lysis buffer (50 mM Tris-HCl, pH 7.5, 150 mM NaCl, 1 mM CaCl_2_, 1% Triton X-100, 0.1% SDS, 0.1% Nonidet P-40, 2 mM PMSF, 1 mM vanadate, 5 *μ*g ml^−1^ Trasylol, 10 *μ*M Pepstatin A, and 10 *μ*M leupeptin). Protein quantity was determined using the Bradford protein assay (Bio-Rad, Hercules, CA, USA), and 50 *μ*g of total protein from each sample was resolved by SDS–PAGE gels and blotted onto polyvinylidene difluoride membranes (Nihon Millipore Ltd, Tokyo, Japan). Membranes were probed overnight with the appropriate primary antibodies at 4 °C followed by incubation with HRP-conjugated secondary antibodies at room temperature for 1 h. Immunoreactive bands were detected using the ECL system (Amersham, Piscataway, NJ, USA). Glyceraldehyde 3-phosphate dehydrogenase (GAPDH) blots served as controls.

### Statistical analysis

Experiments were performed at least three times. Results are expressed as means±s.d. One-way ANOVA was used to determine the overall significance within data groups. A *P*-value of <0.05 was considered significant.

## RESULTS

### Fibronectin stimulates the migration and invasion of A549 human lung adenocarcinoma cells through the activation of FAK

**Figure 1:**
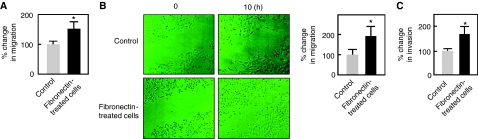
Fibronectin induces the migration and invasion of A549 lung cancer cells. (**A**) A549 cells (5 × 10^4^) suspended in media with or without fibronectin (10 *μ*g ml^−1^) were seeded in the upper chamber of transwell plates (Costar, Cambridge, MA, USA). After 6 h of incubation at 37 °C, cells that migrate to the lower surface of the filters were fixed, stained, and quantified by counting three randomly selected fields under the microscope. (**B**) A549 cells were plated in 35-mm culture dishes and grown to 100% confluence. After generating a single scratch in the monolayer, the cells were washed with PBS, and cultured for another 10 h in media with or without fibronectin. Photographs were taken immediately after wounding and 10 h later. The change in wound width was measured as described in the Materials and methods section. (**C**) A549 cells (5 × 10^4^) suspended in media with or without fibronectin were seeded in Matrigel invasion inserts (BD, Franklin Lakes, NJ, USA). After 24 h in culture, cells that invaded the lower surface of the filters were counted. Values are means±s.d. (*n*=3). Migration and invasion of control cells were set at 100%. Significant difference (^*^) in migration and invasion was set at *P*<0.05.

Interactions between tumour cells and the ECM strongly influence tumour development. Fibronectin, a large multi-domain ECM glycoprotein, regulates many cellular processes, including migration and invasion (Shibata *et al*, 1998; Sawai *et al*, 2005). In human lung adenocarcinoma cells, the involvement of fibronectin in these processes remains to be investigated. Therefore, we sought to characterise the role of fibronectin in the migration and invasion of A549 human lung adenocarcinoma cells. Using *in vitro* transwell migration assays, we found that fibronectin, indeed, induces the migration of A549 lung adenocarcinoma cells (Figure 1A). Furthermore, *in vitro* wound healing assays showed that fibronectin is capable of inducing migration (0.325 *vs* 0.625 mm: 100 *vs* 192% migration) of A549 cells into the wounded area (Figure 1B). As the acquisition of an invasive phenotype by cancer cells is a critical step for tumour progression, we also examined the invasive ability of fibronectin-treated A549 cells through a three-dimensional matrigel-coated filter. As shown in Figure 1C, fibronectin-treated A549 cells had increased ability to traverse matrigel-coated filters compared with untreated control cells. Taken together, these data indicate that fibronectin can stimulate the migration and invasion of lung cancer cells.

**Figure 2:**
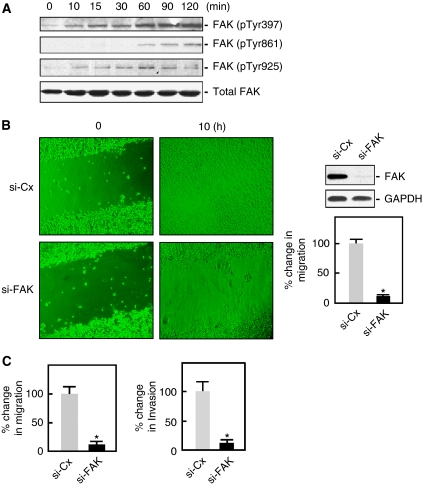
Fibronectin promotes A549 cell migration and invasion through FAK. (**A**) A549 cells were seed in media with or without fibronectin (10 *μ*g ml^−1^) and incubated at 37 °C at the indicated time points. The expression of FAK phosphorylated at Tyr397, Tyr861, and Tyr 925 was then evaluated by western blotting. (**B**) A549 cells transfected with FAK siRNA or control siRNA for 48 h were re-plated in 35-mm dishes. Migration was evaluated as described in Figure 1. Depletion of FAK was determined by western blotting. (**C**) A549 cells transfected with FAK siRNA or control siRNA for 48 h were suspended in media with or without fibronectin and seeded in the upper chamber of transwell plates. The migration of cells to the lower surface of the filters was assessed after 6 h as described in Figure 1. Invasion assay of A549 cells transfected with FAK siRNA or control siRNA for 48 h was also carried out as described in Figure 1. Values are means±s.d. (*n*=3). Migration and invasion of control cells were set at 100%. Significant difference (^*^) in migration and invasion was set at *P*<0.05.

Next, we investigated the molecular mechanism through which fibronectin induces the migration and invasion of lung cancer cells. As previous studies have shown that the ECM ligand–integrin interaction leads to tyrosine phosphorylation of FAK, which is crucial for tumour progression, we sought to examine whether fibronectin could promote tyrosine phosphorylation of FAK in A549 lung adenocarcinoma cells. Indeed, we found that fibronectin significantly enhanced the phosphorylation of FAK at tyrosine 397, tyrosine 861, and tyrosine 925 residues (Figure 2A), suggesting that fibronectin-induced migration and invasion of A549 cells may be a result of FAK activation. To substantiate this finding, we depleted FAK by siRNA treatment and examined the effect of FAK depletion on fibronectin-mediated migration and invasion of A549 cells. As shown in Figure 2B and C, there was a dramatic inhibition of fibronectin-induced migration (0.56 *vs* 0.1 mm: 100 *vs* 18%) and invasion in FAK-depleted A549 cells, suggesting a critical role of FAK activation in these processes.

### FAK-induced A549 migration and invasion occur through Src, ERK, and PI3K/Akt activation

**Figure 3:**
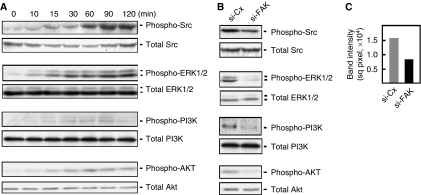
FAK promotes A549 lung cancer cells migration and invasion through Src, ERK, and PI3K/Akt. (**A**) A549 cells were seeded in media with or without fibronectin (10 *μ*g ml^−1^) and incubated at 37 °C at the indicated time points. (**B**) A549 lung cancer cells transfected with FAK siRNA or control siRNA for 48 h were seeded in media with fibronectin and incubated at 37 °C for 2 h. Cell lysates were analysed by western blotting using the indicated antibodies. (**C**) Quantitation of p-Src levels (in square pixels) in FAK siRNA- and control siRNA-treated cells was assessed by densitometric analysis using the NIH Image J software (NIH Image, Bethesda, MD, USA).

To investigate the potential mechanism of FAK signalling in lung cancer cell migration and invasion, we examined the activation of the downstream signalling molecules of integrin. We found that A549 cells treated with fibronectin showed increased phosphorylation of Src, ERK, PI3K, Akt, (Figure 3A), and JNK (data not shown) in a time-dependent manner. Thus, it appears that fibronectin-associated FAK signalling in lung cancer cells occurs through Src, ERK, PI3K, Akt, and JNK. We then examined changes in the activation of these molecules after the depletion of FAK by siRNA in A549 cells. As shown in Figure 3B, the levels of p-Src, p-ERK1/2, p-PI3K, and p-Akt in these cells were reduced. No change in p-JNK was observed (data not shown). Taken together, our results suggest that fibronectin-mediated stimulation of lung cancer cell migration and invasion by FAK is achieved through Src, ERK1/2, and PI3K/Akt signalling pathways.

### FAK promotes A549 migration and invasion of lung cancer cells through cross talk with Src, and Src regulation of ERK and PI3K/Akt

**Figure 4:**
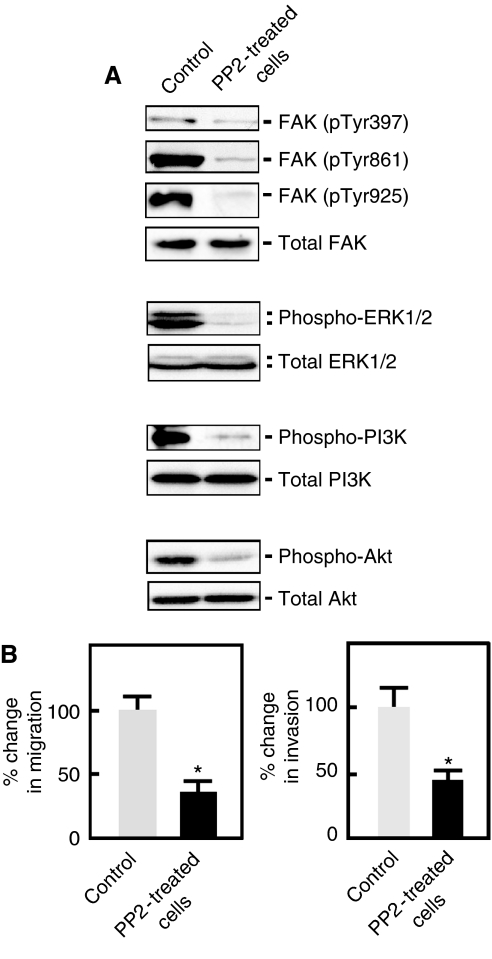
Src is required for the activation of FAK and its downstream targets, ERK and PI3K/AKT during fibronectin-induced migration and invasion. (**A**) A549 cells pretreated with PP2 (20 *μ*M) or DMSO for 48 h were seeded in media containing fibronectin+PP2 or fibronectin+DMSO and incubated at 37 °C for 2 h. Cells were then harvested and analysed by western blotting using the indicated antibodies. (**B**) A549 cells were pretreated with PP2 or DMSO for 48 h. Migration and invasion assays were carried out as described in Figure 2, except for the use of media containing fibronectin+PP2 or fibronectin+DMSO. Values are means±s.d. (*n*=3). Migration and invasion of control cells were set at 100%. Significant difference (^*^) in migration and invasion was set at *P*<0.05.

In many signalling milieus, the FAK–Src interaction serves to control cell motility, but the mechanism underlying its activity is only partly understood. As indicated above, we found that the depletion of FAK caused decreased phosphorylation of Src. To further characterise the relationship between FAK and Src, we also examined the consequence of Src inhibition on the phosphorylation of FAK in A549 cells grown in fibronectin-coated plates. Our results showed that upon treatment of A549 cells with the Src inhibitor, PP2, phosphorylation of FAK at its Tyr397, Tyr861, and Tyr925 residues was noticeably reduced (Figure 4A), indicating that Src also has an important role in the regulation of FAK and its downstream targets. Indeed, FAK-associated phosphorylation of ERK1/2, PI3K, and Akt was also reduced in PP2-treated cells (Figure 4A). In addition, compared with control cells, the migration and invasive ability of A549 cells decreased in the presence of PP2 (Figure 4B). Taken together, these data further suggest that the FAK–Src interaction promotes the invasion and migration of A549 lung cancer cells through the ERK1/2 and PI3K/Akt pathways.

### FAK promotes A549 cell invasion and migration through regulation of MMP9 and calpain-2 expression via ERK1/2

**Figure 5:**
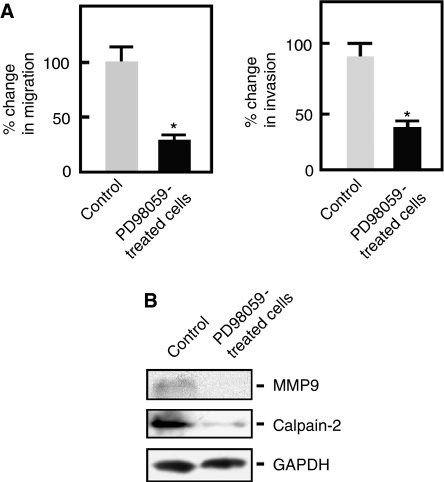
ERK promotes A549 lung cancer cell migration and invasion through MMP9 and calpain-2. (**A**) Migration and invasion assays of A549 cells pretreated with PD98059 (20 *μ*M) or DMSO for 48 h were carried out as described in Figure 4, except that PD98059 was used as treatment instead of PP2. Values are means±s.d. (*n*=3). Migration and invasion of control cells were set at 100%. Significant difference (^*^) in migration and invasion was set at *P*<0.05. (**B**) A549 cells pretreated with PD98059 or DMSO and seeded in media containing fibronectin+PD98059 or fibronectin+DMSO were harvested after 48 h, and analysed by western blotting using the indicated antibodies.

To further examine the involvement of ERK1/2 in FAK signalling during A549 cell migration and invasion, we took advantage of the availability of PD98059, an inhibitor of the ERK1/2 upstream kinase, MEK1/2. As shown in Figure 5A, PD98059 treatment of A549 cells resulted in a significant inhibition of the migration and invasion of these cells, supporting our view that FAK promotes A549 cell invasion and migration through ERK1/2.

As FAK has been suggested to regulate cell migration and invasion by promoting MMP-mediated matrix degradation (Shibata *et al*, 1998) and focal adhesion dynamics (Glading *et al*, 2000; Shao *et al*, 2006), which is regulated by calpain (Glading *et al*, 2000; Huang *et al*, 2004), we next sought to determine whether MMP9 and calpain-2 expression is regulated through ERK1/2. Using western blotting, we found that A549 cells treated with PD98059 showed decreased expression of both MMP9 and calpain-2 (Figure 5B), suggesting that ERK1/2 regulates the expression of these proteins in A549 lung cancer cells. Taken together, our data suggest that FAK promotes A549 cell invasion and migration through ERK1/2-MMP9 and ERK1/2-calpain-2 signalling mechanisms.

### FAK promotes A549 cell invasion and migration through the regulation of MMP9 and RhoA expression via PI3K

**Figure 6:**
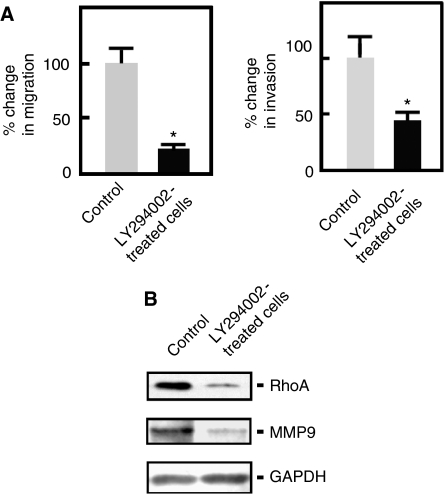
PI3K promotes A549 lung cancer cell migration and invasion through MMP9 and RhoA. (**A**) Migration and invasion assays of A549 cells pretreated with LY294002 or DMSO for 48 h were carried out as described in Figure 4, except that LY294002 was used as treatment instead of PP2. Values are means±s.d. (*n*=3). Migration and invasion of control cells were set at 100%. Significant difference (^*^) in migration and invasion was set at *P*<0.05. (**B**) A549 cells pretreated with LY294002 or DMSO and seeded in media containing fibronectin+LY294002 or fibronectin+DMSO were harvested after 48 h, and analysed by western blotting using the indicated antibodies.

To examine the potential involvement of PI3K in FAK signalling, we examined the effect of the PI3K inhibitor, LY294002, on the migration and invasion of A549 cells. As shown in Figure 6, treatment with LY294002 caused a significant decrease in the migration and invasion of A549 cells, suggesting that FAK-mediated A549 cell invasion and migration involves PI3K signalling. As both MMP-mediated matrix degradation and RhoA-mediated regulation of contraction and retraction forces have been implicated in FAK-induced cell migration and invasion (Lim *et al*, 2008; Luo *et al*, 2009), we further examined the expression of MMP9 and RhoA in A549 cells treated with LY294002. As shown in Figure 6B, both MMP9 and RhoA expression was reduced in cells treated with LY294002, indicating that the expressions of these proteins were regulated through PI3K signalling. Therefore, in addition to ERK1/2-MMP9 and ERK1/2-calpain-2 signalling, FAK also promotes A549 cell invasion and migration through the PI3K/Akt-MMP9 and PI3K/Akt-RhoA pathways.

**Figure 7:**
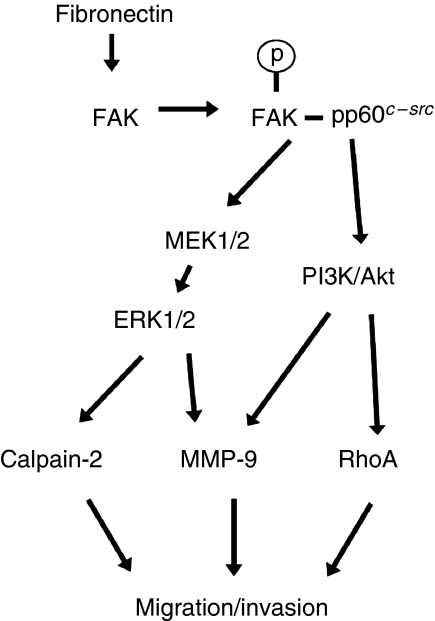
Fibronectin-mediated FAK signalling pathways in lung cancer cell migration and invasion. Fibronectin-stimulated A549 cell migration and invasion is achieved through the activation of FAK, which regulates calpain/MMP-9 and RhoA/MMP9 expression through ERK1/2 and PI3K/Akt signalling, respectively.

To summarise our results, fibronectin-stimulated A549 cell migration and invasion is achieved through the activation of FAK, which regulates calpain/MMP-9 and RhoA/MMP9 expression through ERK1/2 and PI3K/Akt signalling, respectively (Figure 7).

## DISCUSSION

The ECM regulates many cellular events, such as cell differentiation, proliferation, and apoptosis. In many cells, it has been shown that activation of the mitogen-activated protein kinase (MAPK) due to the interaction of integrin with the ECM requires FAK (Sawai *et al*, 2005). Furthermore, integrin receptor binding to ECM proteins seems to activate FAK, which is also involved in the regulation of cellular events, such as cell migration and invasion (Sanders and Basson, 2000; Sawai *et al*, 2005).

Our current findings indicate that the ECM glycoprotein, fibronectin, specifically promotes A549 lung cancer cell migration and invasion. Consistent with previous reports that FAK has a critical role in promoting cell migration and invasion (Mukhopadhyay *et al*, 2005; Zeng *et al*, 2006), we found an increased activation of FAK in A549 cells grown in fibronectin-coated plates. Focal adhesion kinase activation was determined by increased phosphorylation of FAK at its Tyr397, Tyr861, and Tyr925 residues. The relevance of FAK in fibronectin-mediated cell invasion and migration was supported by our data showing that these processes are inhibited after the depletion of FAK by siRNA.

The signalling events downstream of FAK that affect cellular migration and invasion are complex. For example, autophosphorylation of FAK at Tyr397 due to integrin engagement creates high affinity binding for Src, which then gets activated. In turn, activated Src phosphorylates FAK at Tyr397, Tyr861, and Tyr925 residues (Calalb *et al*, 1995, 1996). Focal adhesion kinase activation through Tyr925 phosphorylation and recruitment of GRB2 into the FAK–Src complex lead to enhanced activation of targets, such as ERK1/2 (Schlaepfer and Hunter, 1996). In addition, FAK phosphorylation at Tyr397 mediates its interaction with the SH2 domain of the p85 subunit of PI3K (Chen *et al*, 1996).

To better understand the mechanism by which FAK regulates fibronectin-induced cell migration and invasion, we examined the expression and activity of the downstream targets of integrin receptors. Consistent with previous findings in other cell types (Sanders and Basson, 2000; Schmitz *et al*, 2005; Vulin *et al*, 2005; Stulic *et al*, 2007), we found an enhanced activation of Src, ERK, PI3K, and Akt in A549 lung cancer cells. The role of FAK in the activation of these effectors was noted through their downregulated activation after the depletion of FAK. On the basis of our results, it appears that FAK promotes fibronectin-mediated lung cancer metastasis through the activation of Src, ERK, PI3K, and Akt. Although JNK has been implicated in FAK signalling that promotes cell invasion and migration (Hauck *et al*, 2001; Hsia *et al*, 2003), its apparent lack of involvement in fibronectin-mediated FAK signalling in lung cancer cells may indicate distinct responses by different cell types to different ligands. Alternatively, JNK may be activated through other direct or indirect integrin signalling pathways.

In this study, we further examined whether Src affects FAK activation, and also studied the manner by which the interaction between FAK and Src regulates their downstream targets. Upon treatment of A549 cells with the PP2 Src inhibitor, we found reduced activation of FAK as evidenced by reduced phosphorylation at its Tyr397, Tyr861, and Tyr925 residues. We also showed the inhibition of ERK1/2 and PI3K/AKT in PP2-treated cells. Thus, it seems that FAK promotes A549 lung cancer cell migration and invasion in concert with Src and subsequent activation of the ERK and PI3K/Akt signalling pathways.

The involvement of FAK-stimulated ERK1/2 signalling in fibronectin-induced A549 lung cancer cell migration and invasion was supported by our observation that these processes are inhibited in cells treated with PD98059, an inhibitor of the ERK1/2 upstream kinase, MEK. Studies in ovarian carcinoma cells have shown that FAK and ERK1/2 are important for fibronectin-stimulated invasiveness and MMP9 secretion by these cells (Shibata *et al*, 1998). Moreover, it was found that the MMP9 gene promoter is partially regulated through activation of the ERK1/2 pathway (Gum *et al*, 1997). Thus, in an effort to understand the specific role of ERK1/2 in A549 cell migration and invasion, we examined the expression of MMP9 in A549 cells treated with PD98059. Our finding that these cells have decreased MMP9 expression points to the involvement of this protein in A549 cell migration and invasion. This is consistent with the fact that MMP9 is involved in the degradation of matrix components and thus supports the invasion of tumour cells through basement membrane barriers. Therefore, it appears that FAK promotes A549 lung cancer cell invasion through the ERK/MMP9 pathway.

Previous studies have shown that calpain proteases are required for rear de-adhesion during productive motility whether initiated by adhesion-related signals or growth factors (Glading *et al*, 2000). For example, EGF-induced cell de-adhesion and migration require the activation of the calpain-2 isoform by MAPK at the cell membrane (Shao *et al*, 2006). Our hypothesis was that calpain-2 promotes cell migration through ERK signalling. Indeed, treatment of A549 lung cancer cells with PD98059 caused a decrease in the expression of calpain-2. Taken together, our findings support the view that fibronectin induces the activation of FAK which then activates both the ERK1/2/MMP9 and ERK1/2/calpain-2 pathways to promote lung cancer cell invasion and migration, respectively.

There have been suggestions that activation of PI3K regulates the migration and invasion of various cancer cells (Reiske *et al*, 1999; Zeng *et al*, 2006). Despite these findings, the specific role of PI3K in controlling lung cancer cell migration and invasion, particularly in relation to FAK signalling, has been elusive. In this study, we found that PI3K lies downstream of FAK. Inhibition of PI3K in A549 lung cancer cells by LY294002, showed decreased migration and invasive ability of A549 cells, suggesting that PI3K is also involved in the fibronectin-induced FAK signalling. As with ERK, PI3K has been implicated in the activation of MMP-9 secretion (Han *et al*, 2006), and as with calpain, RhoA regulates the contraction and retraction forces that are required for cell motility (Raftopoulou and Hall, 2004). In this study, we examined the potential signalling between PI3K and MMP9 or RhoA in A549 cells exposed to fibronectin and found that MMP9 and RhoA expression decreased upon LY294002 treatment, suggesting that FAK further promotes A549 lung cancer cell invasion and migration through PI3K/MMP9 and PI3K/RhoA signalling mechanisms.

Although our RhoA finding contradicts the observation of Chen *et al* (2002), it is consistent with the finding of Amano *et al* (1996) who reported that RhoA could increase the level of phosphorylated myosin, which crosslinks actin filaments and generates contractile forces, promoting movement of the cell body and facilitating cell rear detachment. The difference in our finding from that of Chen *et al* (2002) may be due to the effect of ROCK, which has been shown to positively regulate cell motility together with RhoA, but was also found to negatively affect cell motility depending on cellular conditions (Kimura *et al*, 1996; Maekawa *et al*, 1999). Nevertheless, our study provides the first indication that fibronectin-induced FAK activation stimulates cell migration and invasion through PI3K/MMP-9 and PI3K/RhoA signalling.

In summary, we provide compelling evidence that fibronectin-induced A549 cell migration and invasion occurs through the activation of FAK, which then regulates MMP-9 expression through ERK or PI3K, as well as calpain-2 and RhoA expression through ERK and PI3K, respectively. A model for these fibronectin-mediated FAK signalling mechanisms is shown in Figure 7. Certainly, these signalling pathways may have critical roles in lung cancer metastasis. Our findings also offer new insights into the potential therapeutic significance of FAK in lung cancer.
